# Face‐off: Novel depredation and nest defense behaviors between an invasive and a native predator in the Greater Everglades Ecosystem, Florida, USA

**DOI:** 10.1002/ece3.8639

**Published:** 2022-02-19

**Authors:** Andrea F. Currylow, Matthew F. McCollister, Gretchen E. Anderson, Jillian M. Josimovich, Austin L. Fitzgerald, Christina M. Romagosa, Amy A. Yackel Adams

**Affiliations:** ^1^ U.S. Geological Survey Fort Collins Science Center ‐ South Florida Field Station in Everglades National Park Homestead Florida USA; ^2^ National Park Service Big Cypress National Preserve Ochopee Florida USA; ^3^ Present affiliation: U.S. Fish and Wildlife Service Avon Park Air Force Range Avon Park Florida USA; ^4^ Department of Wildlife Ecology and Conservation University of Florida Gainesville Florida USA; ^5^ U.S. Geological Survey Fort Collins Science Center Fort Collins Colorado USA

**Keywords:** bobcat, Burmese python, intraguild predation, nest depredation, reptile–mammal interaction

## Abstract

We describe several photo‐documented novel interactions between intraguild predators in southern Florida—the native bobcat (*Lynx rufus*) and the invasive Burmese python (*Python bivittatus*). Over several days we documented a bobcat's depredation of an unguarded python nest and subsequent python nest defense behavior following the return of both animals to the nest. This is the first documentation of any animal in Florida preying on python eggs, and the first evidence or description of such antagonistic interactions at a python nest.

## Graphical abstract

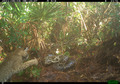

Bobcats (*Lynx rufus*) have broad diets; they hunt and scavenge a variety of birds, small and mid‐sized mammals, and reptiles (Jones & Smith, 1979; Maehr & Brady, 1986; Tewes et al., 2002). In Florida, birds make up approximately 16% of bobcat prey items (Maehr & Brady, 1986) and their diets likely include eggs within nests (see Malone et al., 2019). Indeed, in Georgia they have been documented predating Northern bobwhite (*Colinus virginianus*) nests, with eggs comprising up to 19% of their diet in Georgia (Schoch, 2003; Staller et al., 2005). Although there are few reports of bobcats targeting reptile nests in Florida, up to ~2% of seasonal marine turtle nest mortality has been attributed to bobcat depredation (Lindborg et al., 2016; Martin et al., 2005).

The Burmese python (*Python bivittatus*) is an invasive reptile in the Greater Everglades Ecosystem, disrupting trophic relationships, including the near extirpation of mammals from Everglades National Park (Dorcas et al., 2012; Dove et al., 2012; McCleery et al., 2015; Sovie et al., 2016). Nest predators of pythons are almost completely unknown, with only the Bengal monitor (*Varanus bengalensis*) documented to prey on python eggs (Bhupathy & Vijayan, 1989; Dorcas & Willson, 2011). This is likely a consequence of female Burmese python nest attendance behaviors serving to both defend and brood (via shivering thermogenesis) the clutch (Ramesh & Bhupathy, 2010; Shine, 1988; Snow et al., 2010). Despite nest attendance, there have been no records of a python defending a nest from likely predators such as coyotes (*Canis latrans*), skunks (members of Mephitidae family), raccoons (*Procyon lotor*), mongooses (members of Herpestidae family), or monitor lizards (Minton & Minton, 1973). However, the dearth of information on wild python nesting habits is unsurprising considering their crypsis abilities (e.g., Nafus et al., 2020). Additionally, due to the deleterious impacts on native Florida ecosystems, nearly all invasive pythons discovered are immediately removed and, therefore, information on their vital rates and behaviors remains understudied.

Documented predators of adult bobcats are few but include mountain lions (*Puma concolor*), wolves (*Canis lupus*), coyotes, and, in Florida, Burmese pythons and presumably American alligators (*Alligator mississippiensis*; Gipson & Kamler, 2002; Hass, 2009; Harveson et al., 2000; Snow et al., 2007; Shores et al., 2019). Similarly, only a few Florida predators are capable of preying on adult‐sized Burmese pythons, but the list does include bobcats along with Florida black bears (*Ursus americanus floridanus*), Florida panther (*P*. *c*. *coryi*), American alligators, and conceivably American crocodiles (*Crocodylus acutus*; Godfrey et al., 2021; McCollister et al., 2021; Smith et al., 2016; Snow et al., 2006; Reed & Rodda, 2009).

On June 01, 2021, we deployed a wildlife surveillance camera (still‐photograph movement triggered model: Hyperfire 2, Reconyx, Holmen, Wisconsin, USA) at the nest site of a large (424.0 cm) adult female Burmese python that was telemetered as part of another study in Big Cypress National Preserve, Florida, USA. When we returned to the nest site on June 04, 2021, we discovered that the python nest had been depredated and examination of the camera's memory card implicated a male bobcat. Although python movements are not fast enough to trigger the surveillance camera, bobcat movements are. We found that the same day we deployed the camera, a male bobcat appeared in the camera frame, along with an unguarded python nest. The brooding python apparently departed the nest, leaving her eggs visibly exposed on the ground surface. Using photo metadata, we determined the python was last visible in the frame when we initiated the camera surveillance on June 01, 2021 at 10:35 h. (Figure 1a). Later that day at 17:12 h, the bobcat first appeared on camera (Figure 1b).

**FIGURE 1:**
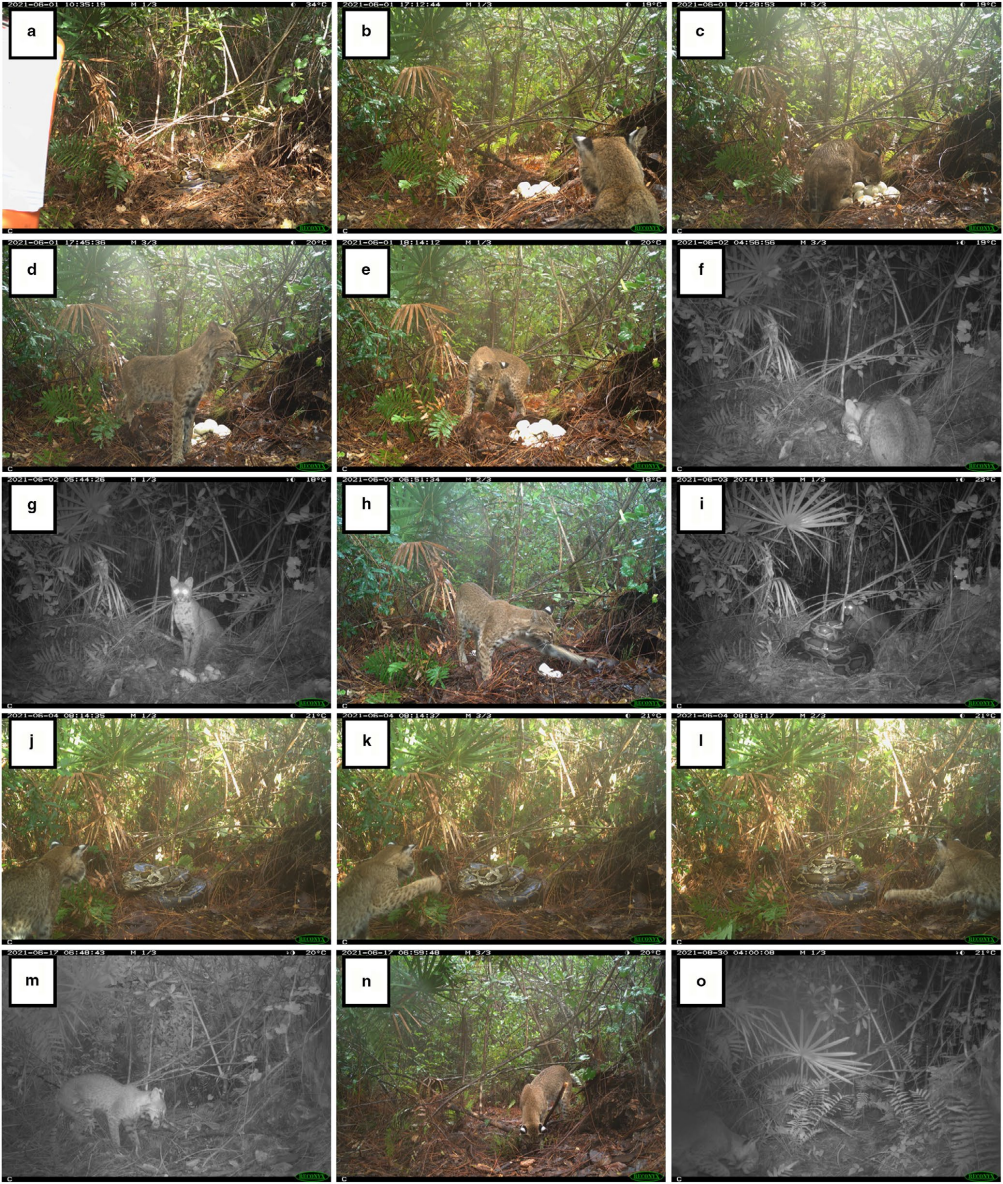
Selected photo sequence at a Burmese python (*Python bivittatus*) nest site from June 01, 2021–August 30, 2021, starting at top left (a) and ending at bottom right (o)—camera deployment initiation photograph with clipboard where python is seen brooding her nest in center of frame (a); bobcat (*Lynx rufus*) discovers unguarded nest (b) and proceeds to depredate, cache, and uncover the eggs over several days (c–h); the bobcat returns to find the female python back on the nest (i) and later proceeds to swipe at the snake (j–l); post‐nest salvage attempt by biologists, the bobcat returns to scavenge discarded, inviable eggs over several weeks (m–o). Entire photo sequence can be viewed in MP4 video format through the ScienceBase Catalog at: https://doi.org/10.5066/P97ZDQHY (Currylow et al., 2022). Photographs were captured in Big Cypress National Preserve within the Greater Everglades Ecosystem, Florida, USA

Over the course of two days, a bobcat (determined to be the same individual based on pelage pattern, general appearance, activity, and location) can be seen repeatedly approaching the unguarded nest and consuming, trampling, caching, and uncovering the eggs (Table 1; Figure 1b–h). The next image of the python brooding the nest is the evening of 02 June when the bobcat again returns, triggering the camera at 17:30 h. At that time, the bobcat can be seen moving around the brooding Burmese python but not closely approaching or interacting with the large snake. The next night (03 June), the bobcat returns, and the two predators can be seen facing each other (Figure 1i), but the bobcat again exits the camera's field of view without obviously provoking the python. On the morning of 04 June, the bobcat again returns to the nest site, triggering the camera to reveal that the python had apparently struck at the bobcat. The strike was determined from the first photos in that sequence, which captures the python's head at striking distance from the rest of her coils near the bobcat's feet. The subsequent photo sequence shows the python back atop her coils, facing the bobcat, which can then be seen swiping at the python from the left side of the frame, then moving to the right as the python visually tracks the bobcat (Figure 1j–l). From the right, the bobcat advances and again swipes at the python but no physical contact is apparent. The final photos in that sequence are of the bobcat flanking the Burmese python.

**TABLE 1:** Timeline of photo‐documented reciprocal intraguild predator interaction (bobcat, *Lynx rufus*, and Burmese python, *Python bivittatus*) at a python nest site in the Greater Everglades Ecosystem, Florida, USA

Date	Time of day	Activity description
2021‐06‐01	10:35	Biologists deploy camera. Python guarding nest, Figure 1a
	17:12	Bobcat first seen at unguarded nest, Figure 1b
	17:28	Bobcat begins to consume eggs, Figure 1c
	17:51–18:20	Bobcat repeatedly exits field of view and returns to nest, Figure 1d–e
	18:24–18:29	Bobcat consumes eggs, walks around nest. Exits field of view
	19:24–19:33	Bobcat returns, inspects nests, exits field of view
	20:59–21:09	Bobcat returns and inspects nest. Exits field of view (images switch to night mode)
	23:27–23:44	Bobcat returns to, inspects, and tramples nest. Exits field of view
2021‐06‐02	01:04	Bobcat returns and consumes eggs (images in night mode)
	01:06–03:26	Unknown activity but eggs heavily disturbed
	03:26	Bobcat exits field of view
	04:54–05:06	Bobcat returns and consumes eggs, Figure 1f. Exits field of view
	05:40–05:51	Bobcat returns. Figure 1g. Exits field of view
	06:29–06:42	Bobcat returns and inspects nest (images switch to day mode)
	06:51	Bobcat begins caching eggs, Figure 1h
	06:55	Bobcat exits field of view
	08:09	Bobcat returns and begins uncovering/consuming eggs
	09:00	Bobcat exits field of view
	11:18–11:54	Bobcat repeatedly returns, digs at, and consumes eggs. Exits field of view
	12:21–12:22	Bobcat returns and uncovers eggs. Exits field of view
	17:30–17:53	Python back on nest, bobcat returns but does not approach, Figure 1i. (images in night mode). Python shifts position, bobcat exits field of view for >24 h
2021‐06‐03	20:39	Python shifted position, bobcat returns in foreground, python visibly inhales and exhales (images in night mode)
	20:41	Bobcat appears in background facing python. Bobcat exits field of view
2021‐06‐04	08:14–08:17	Bobcat returns, python apparently struck at bobcat. Python faces bobcat. Bobcat repeatedly swipes at python while moving around nest. Figure 1j‐l
	08:17	Final photo of interaction—bobcat flanks python
	10:27–11:34	Biologists arrive, move python from nest, discover nest has been depredated
2021‐06‐15	10:25–11:12	Biologists return and retrieve potentially viable eggs and reset camera
2021‐06‐17	06:47–07:04	Bobcat returns to nest site and scavenges inviable eggs left by biologists. Figure 1m,n (images switch to day mode)
2021‐07‐01	05:29	Bobcat again returns to nest site and walks through frame
2021‐08‐30	04:00	Bobcat returns to nest site but remains near edge of frame, Figure 1o
2021‐09‐18	09:40	Biologists retrieve camera

Just over two hours later, our team of biologists are captured on camera assessing the Burmese python and noting the poor condition of the nest. The team left the area, and no further photos were captured until the team returned on 15 June to reset the camera. At that time, the python was on and brooding the damaged nest. We moved the animal from the nest to salvage eggs for laboratory incubation. We documented 42 inviable or destroyed eggs and 22 damaged but potentially viable eggs weighing a total of 5.5 kg. We incubated the 22 potentially viable eggs at 31℃ in commercially available, circulated‐air egg incubators with moistened horticultural perlite substrate until each egg showed obvious signs of decay (up to 26 days later); none hatched from this nest though others from other project nests did under the same conditions. We dissected each decaying egg to confirm its status, all of which contained either desiccated, hardened yolk or were putrid and infested with insect larvae.

Although no further photos captured the python at the nest site after 15 June, over the next several weeks the camera captured a bobcat investigating the site and scavenging the destroyed eggs and eggshells left by the biologists (Figure 1m–o). Also captured on camera during those several weeks were Virginia opossum (*Didelphis virginiana*) and hispid cotton rat (*Sigmodon hispidus*; see Currylow et al., 2022), all of which are known to be python prey (Reed & Rodda, 2009; Snow et al., 2007).

Using published average weights, the photos, and accounting for sex, we estimate the bobcat to have weighed approximately 9 kg at the time of the reciprocal intraguild interaction. Although previous work has shown that bobcats can successfully prey on adult Burmese pythons in the Greater Everglades Ecosystem (McCollister et al., 2021), the adult male python in that case was much smaller (310.0 cm total length and 14.7 kg) than the female we described herein (see below). Considering the size and varied diet of Burmese pythons in Florida, such bold behavior by the bobcat around a large python had the potential to be fatal for the bobcat if the python had been interested in feeding.

Brooding female pythons generally do not feed until they abandon the nest approximately 3–13 days before hatching (Hanslowe et al., 2016; Ramesh & Bhupathy, 2010; Wolf et al., 2016). In April, the prenesting female python measured 424.0 cm total length and weighed 54.3 kg. On the day of camera reset in June, the brooding animal's postnesting weight was 38.8 kg, presumably losing weight due to the combination of egg‐laying and brooding‐time anorexia (Wolf et al., 2016). Indeed, the postnesting python was found with an obvious bolus of fecal matter near the end of the gastrointestinal tract in July 2021 and had gained 22.5 kg (nearly 60%) since the month prior. During handling, the animal expelled some of the fecal material, chiefly comprising hair with a few small deer hooves and hoof sheaths from a fawn or young white‐tailed deer (*Odocoileus virginianus*), a species declining in the region (Cherry et al., 2019).

Although it has long been presumed that python nest attendance behavior serves to guard against nest predators, observations of active nest defense in the wild are almost never observed or verified (Shine, 1988). Because pythons may only briefly leave their nests during brooding (Minton & Minton, 1973), opportunities for successful nest predation are limited and likely rare. Herein we describe, to our knowledge, the first recorded instance of a Burmese python actively defending a nest and the first record of a bobcat depredating a python nest.

## CONFLICT OF INTEREST

None declared.

## AUTHOR CONTRIBUTIONS

**Andrea F. Currylow:** Conceptualization (lead); Supervision (equal); Writing – original draft (lead). **Matthew F. McCollister:** Conceptualization (supporting); Resources (equal); Writing – review & editing (equal). **Gretchen E. Anderson:** Investigation (equal); Writing – original draft (supporting); Writing – review & editing (equal). **Jillian M. Josimovich:** Project administration (lead); Writing – review & editing (equal). **Austin L. Fitzgerald:** Investigation (equal); Writing – review & editing (equal). **Christina M. Romagosa:** Resources (equal); Supervision (equal); Writing – review & editing (equal). **Amy A. Yackel Adams:** Conceptualization (supporting); Supervision (equal); Writing – review & editing (equal).

## Data Availability

The data that support the findings of this study are available within this article (Figure 1) and can also be viewed in their entirety available in the ScienceBase Catalog at: 10.5066/P97ZDQHY (Currylow et al., 2022).

## References

[ece38639-bib-0001] Bhupathy, S. , & Vijayan, V. S. (1989). Status, distribution, and general ecology of the Indian Python, *Python molurus molurus*. Linn. in Keoladeo National Park, Bharatpur, Rajasthan, India. The Journal of the Bombay Natural History Society, 86, 381–387.

[ece38639-bib-0002] Cherry, M. J. , Crawford, D. A. , Engebretsen, K. N. , Abernathy‐Conners, H. N. , Ellsworth, W. H. , Stiffler, L. L. , Bled, F. , Garrison, E. P. , Miller, K. V. , Warren, R. J. , Conner, L. M. , & Chandler, R. B. (2019). South Florida Deer Study: Final Report to the Florida Fish and Wildlife Conservation Commission (pp. 294). Warnell School of Forestry and Natural Resources, University of Georgia.

[ece38639-bib-0003] Currylow, A. F. , Anderson, G. E. & Yackel Adams, A. A. (2022). Photo‐documented sequences from 01 Jun 2021‐30 Aug 2021 showing novel interactions between intraguild predators in southern Florida, USA, bobcat and Burmese python: U.S. Geological Survey data release. 10.5066/P97ZDQHY

[ece38639-bib-0004] Dorcas, M. E. , & Willson, J. D. (2011). Invasive pythons in the United States: Ecology of an introduced predator. University of Georgia Press.

[ece38639-bib-0005] Dorcas, M. E. , Willson, J. D. , Reed, R. N. , Snow, R. W. , Rochford, M. R. , Miller, M. A. , Meshaka, W. E. , Andreadis, P. T. , Mazzotti, F. J. , Romagosa, C. M. , & Hart, K. M. (2012). Severe mammal declines coincide with proliferation of invasive Burmese pythons in Everglades National Park. Proceedings of the National Academy of Sciences of the United States of America, 109, 2418–2422. 10.1073/pnas.1115226109 22308381 PMC3289325

[ece38639-bib-0006] Dove, C. J. , Reed, R. N. , & Snow, R. W. (2012). Consumption of bird eggs by invasive Burmese pythons in Florida. IRFC Reptiles & Amphibians, 19, 64–66. 10.17161/randa.v19i1.13848

[ece38639-bib-0007] Gipson, P. S. , & Kamler, J. F. (2002). Bobcat killed by a Coyote. Southwestern Naturalist, 47, 511–513. 10.2307/3672519

[ece38639-bib-0008] Godfrey, S. , Squires, M. , Metzger, E. , Mazzotti, F. , Darline, R. , & Muhly, R. (2021). *Crocodylus acutus* (American Crocodile). Interspecific interaction. Herpetological Review, 52, 641–642.

[ece38639-bib-0009] Hanslowe, E. B. , Falk, B. G. , Collier, M. A. , Josimovich, J. M. , Rahill, T. A. , & Reed, R. N. (2016). First record of invasive Burmese python oviposition and brooding inside an anthropogenic structure. Southeastern Naturalist, 15, 103–107. 10.1656/058.015.sp809

[ece38639-bib-0010] Harveson, L. A. , Tewes, M. E. , Silvy, N. J. , & Rutledge, J. (2000). Prey Use by mountain lions in southern Texas. Southwestern Naturalist, 45, 472–476. 10.2307/3672595

[ece38639-bib-0011] Hass, C. C. (2009). Competition and coexistence in sympatric bobcats and pumas. Journal of Zoology, 278, 174–180. 10.1111/j.1469-7998.2009.00565.x

[ece38639-bib-0012] Jones, J. H. , & Smith, N. S. (1979). Bobcat density and prey selection in central Arizona. Journal of Wildlife Management, 43, 666–672. 10.2307/3808745

[ece38639-bib-0013] Lindborg, R. , Neidhardt, E. , Witherington, B. , Smith, J. R. , & Savage, A. (2016). Factors influencing loggerhead (*Caretta caretta*) and green turtle (*Chelonia mydas*) reproductive success on a mixed use beach in Florida. Chelonian Conservation and Biology, 15, 238–248.

[ece38639-bib-0014] Maehr, D. S. , & Brady, J. R. (1986). Food habits of bobcats in Florida. Journal of Mammalogy, 67, 133–138. 10.2307/1381009

[ece38639-bib-0015] Malone, K. M. , Jones, H. H. , Betancourt, A. M. , Terhune Ii, T. M. , & Sieving, K. E. (2019). Video documentation of predators and nest defense at Bachman's sparrow nests. Avian Conservation and Ecology, 14, art. 6.

[ece38639-bib-0016] Martin, R. , Engeman, R. , Smith, H. , Stahl, M. , & Constantin, B. (2005). Cheloniidae (marine turtle) bobcat nest predation. Herpetological Review, 36, 56–57.

[ece38639-bib-0017] McCleery, R. A. , Sovie, A. , Reed, R. N. , Cunningham, M. W. , Hunter, M. E. , & Hart, K. M. (2015). Marsh rabbit mortalities tie pythons to the precipitous decline of mammals in the Everglades. Proceedings of the Royal Society B: Biological Sciences, 282(1805), 20150120. 10.1098/rspb.2015.0120 PMC4389622 25788598

[ece38639-bib-0018] McCollister, M. , Josimovich, J. , Fitzgerald, A. , Jansen, D. , & Currylow, A. F. (2021). Native mammalian predators can predate adult Burmese pythons in Florida. Southeastern Naturalist, 20, N55–N59. 10.1656/058.020.0205

[ece38639-bib-0019] Minton, S. A. , & Minton, M. R. (1973). Giant Reptiles. C. Scribner's Sons.

[ece38639-bib-0020] Nafus, M. G. , Mazzotti, F. J. , & Reed, R. N. (2020). Estimating detection probability for Burmese pythons with few detections and zero recaptures. Journal of Herpetology, 54, 24–30. 10.1670/18-154

[ece38639-bib-0021] Ramesh, C. , & Bhupathy, S. (2010). Breeding biology of *Python molurus molurus* in Keoladeo National Park, Bharatpur, India. The Herpetological Journal, 20, 157–163.

[ece38639-bib-0022] Reed, R. N. , & Rodda, G. H. (2009). Giant constrictors: Biological and management profiles and an establishment risk assessment for nine large species of Pythons, Anacondas, and the Boa Constrictor. Report no. 2009–1202.

[ece38639-bib-0023] Schoch, B. N. (2003). Diet, age, and reproduction of mesomammalian predators in response to intensive removal during the quail nesting season. Master's Thesis. Athens, Georgia, USA: University of Georgia.

[ece38639-bib-0024] Shine, R. (1988). Parental care in reptiles. In C. Gans & R. Huey (Eds.), Biology of the reptilia, Vol. 16 (pp. 275–329). A.R. Liss.

[ece38639-bib-0025] Shores, C. R. , Dellinger, J. A. , Newkirk, E. S. , Kachel, S. M. , & Wirsing, A. J. (2019). Mesopredators change temporal activity in response to a recolonizing apex predator. Behavioral Ecology, 30, 1324–1335. 10.1093/beheco/arz080

[ece38639-bib-0026] Smith, B. J. , Cherkiss, M. S. , Hart, K. M. , Rochford, M. R. , Selby, T. H. , Snow, R. W. , & Mazzotti, F. J. (2016). Betrayal: Radio‐tagged Burmese pythons reveal locations of conspecifics in Everglades National Park. Biological Invasions, 18, 3239–3250. 10.1007/s10530-016-1211-5

[ece38639-bib-0027] Snow, R. W. , Brien, M. L. , Cherkiss, M. S. , Wilkins, L. , & Mazzotti, F. J. (2007). Dietary habits of the Burmese python, *Python molurus bivittatus,* in Everglades National Park, Florida. Herpetological Bulletin, 101, 5–7.

[ece38639-bib-0028] Snow, R. , Oberhoffer, L. , & Mazzotti, F. (2006). *Alligator mississippiensis* (American alligator), Feeding. Herpetological Review, 37, 80–81.

[ece38639-bib-0029] Snow, R. W. , Wolf, A. J. , Greeves, B. W. , Cherkiss, M. S. , Hill, R. , & Mazzotti, F. J. (2010). Thermoregulation by a brooding Burmese python (*Python Molurus Bivittatus*) in Florida. Southeastern Naturalist, 9, 403–405. 10.1656/058.009.0215

[ece38639-bib-0030] Sovie, A. R. , McCleery, R. A. , Fletcher, R. J. , & Hart, K. M. (2016). Invasive pythons, not anthropogenic stressors, explain the distribution of a keystone species. Biological Invasions, 18, 3309–3318. 10.1007/s10530-016-1221-3

[ece38639-bib-0031] Staller, E. , Palmer, W. , Carroll, J. , Thornton, R. P. , & Sisson, D. C. (2005). Identifying predators at northern bobwhite nests. The Journal of Wildlife Management, 69, 124–132. 10.2193/0022-541X(2005)069<0124:IPANBN>2.0.CO;2

[ece38639-bib-0032] Tewes, M. E. , Mock, J. M. , & Young, J. H. (2002). Bobcat predation on quail, birds, and mesomammals. In S. J. DeMaso , W. P. Kuvlesky Jr. , F. Hernandez , & M. E. Berger (Eds.), Quail V: Proceedings of the Fifth National Quail Symposium (pp. 65–70). Texas Parks and Wildlife Department.

[ece38639-bib-0033] Wolf, A. J. , Walters, T. M. , Rochford, M. R. , Snow, R. W. , & Mazzotti, F. J. (2016). Incubation temperature and sex ratio of a *Python bivittatus* (Burmese python) clutch hatched in Everglades National Park, Florida. Southeastern Naturalist, 15, 35–39.

